# Microbiome-Metabolomics Analysis Investigating the Impacts of Dietary Starch Types on the Composition and Metabolism of Colonic Microbiota in Finishing Pigs

**DOI:** 10.3389/fmicb.2019.01143

**Published:** 2019-05-29

**Authors:** Miao Yu, Zhenming Li, Weidong Chen, Ting Rong, Gang Wang, Xianyong Ma

**Affiliations:** Institute of Animal Science, Guangdong Academy of Agricultural Sciences, State Key Laboratory of Livestock and Poultry Breeding, Key Laboratory of Animal Nutrition and Feed Science in South China, Ministry of Agriculture, Guangdong Public Laboratory of Animal Breeding and Nutrition, Guangdong Engineering Technology Research Center of Animal Meat Quality and Safety Control and Evaluation, Guangzhou, China

**Keywords:** colon, metabolic profiles, microbiota, pigs, starch sources

## Abstract

The present study used a combination of 16S rRNA MiSeq sequencing strategy and gas chromatograph time of flight mass spectrometer (GC-TOF/MS) technique to investigate the effects of starch sources on the colonic microbiota and their metabolites in finishing pigs. A total of 72 crossbred barrows were allocated to three different experimental diets with eight replicates and three pigs per replicate. The diet types included tapioca starch (TS), corn starch (CS), and pea starch (PS) (amylose/amylopectin were 0.11, 0.25, and 0.44, respectively). Results showed that the PS diet markedly increased (adjusted *P* < 0.05) the abundance of short-chain fatty acids (SCFAs) and lactate producers, such as *Lactobacillus*, *Prevotella*, *Faecalibacterium*, and *Megasphaera*, while decreased (adjusted *P* < 0.05) the abundance of *Escherichia coli* when compared with the TS diet. The metabolomic and biochemistry analyses demonstrated that the PS diet increased (adjusted *P* < 0.05) the concentrations of organic acids (acetate, propionate, butyrate, valerate, and lactate) and some macronutrients (sugars and long-chain fatty acids), and decreased (adjusted *P* < 0.05) the amino acids and their derivatives (leucine, glycine, putrescine, cadaverine, skatole, indole, and phenol) when compared with the TS diet. Additionally, Spearman’s correlation analysis revealed that the changes in the colonic metabolites were associated with changes in the microbial composition. Correlatively, these findings demonstrated that the different dietary starch types treatment significantly altered the intestinal microbiota and metabolite profiles of the pigs, and dietary with higher amylose may offer potential benefits for gut health.

## Introduction

Starch, acts as a major energy source of the daily diet and is the largest fraction among human and monogastric animal diets (Yin et al., 2010). Dietary starches from different sources can affect digestion and absorption at different rates and to different extents, depending on the physicochemical properties of the starch (Giuberti et al., 2015). Moreover, the rate, extent, and site at which the starch is degraded can cause different physiological impacts on the physiological function of the gastrointestinal system and the gut health of the host (Giuberti et al., 2015; Metzler-Zebeli et al., 2018). Generally, starch contains two types of molecules, amylose and amylopectin (Tester et al., 2004), and the digestion rate of starch is largely dependent on the proportion of amylose to amylopectin that the starch molecule contains. Amylopectin is recognized to be rapidly digested because its branched structure provides multiple sites for enzymatic hydrolysis by amylase. In contrast, amylose is a more linear glucose polymer and is not degraded in the small intestine by either pancreatic α-amylase or brush border disaccharide hydrolases. It then reaches the large intestine where it can be fermented by the resident microbiota, providing a carbon source and energy for the bacteria (Englyst et al., 1992; Lafiandra et al., 2014). Starch with a higher proportion of amylose can decrease endogenous digestibility in the small intestine and subsequently increase the digesta mass reaching the large intestine for microbial fermentation (Topping et al., 1997). Alterations in substrate degradation by intestinal microbiota can induce changes in the microbiota as well as the metabolic end products of microbial degradation (Fouhse et al., 2015; Yin et al., 2018a). Accumulating evidence has indicated that diets containing starch with a higher content of amylose can increase distal digesta mass, short-chain fatty acid (SCFA) concentration, and commensal microbial populations in the gut, including *Bifidobacterium* spp. and *Lactobacillus* (Bird et al., 2007, 2009; Regmi et al., 2011). Increases in SCFAs, especially butyrate, have many important nutritional and physiological effects on maintaining intestinal health (Newman et al., 2018). However, until now, information on the effects of different starch sources on other microbial metabolites in the gut is limited, and the relationships among starch sources, microbial community, and microbial activity is not clearly understood.

Therefore, diets containing three purified starches with clear differences in the ratio between amylose and amylopectin, were fed to pigs to test the hypothesis that dietary starches with high amylose can exert different impacts on the gut bacterial community and microbial metabolites in pigs. The present study used a 16S rRNA MiSeq sequencing strategy and combined with gas chromatograph time of flight mass spectrometer (GC-TOF/MS) technique to investigate the effects of starch sources (tapioca starch, corn starch, and pea starch) on the colonic microbial composition and microbial metabolites in pigs.

## Materials and Methods

### Ethics Statement

The experimental proposals and procedures for the care and treatment of the pigs were approved by the Animal Care and Use Committee of Guangdong Academy of Agricultural Sciences (Authorization No. GAASIAS-2016-017).

### Animals, Diets, and Sampling

Seventy-two crossbred (Duroc × Landrace × Large White) growing barrows were randomly allocated to three different experimental diets based on their body weight (BW, 77 ± 0.52 kg). Each dietary group consisted of eight pens (replicates), with three pigs per pen. Pigs in the three treatments were fed tapioca starch (TS group), corn starch (CS group), or pea starch (PS group), respectively, as their dietary starch sources. The ratio of amylose to amylopectin of the three diets were 0.11, 0.25, and 0.44, respectively. The experimental diets were formulated to meet or exceed the nutrient recommendations of the National Research Council (NRC) (Table 1) (NRC, 2012). The diets and water were provided with *ad libitum* throughout the 40-day feeding trial. The feed consumption per pen was recorded every day to calculate average daily feed intake (ADFI). The BWs of all pigs were recorded at the beginning and the end of the study period to determine average daily gain (ADG).

**Table 1:** Feed ingredient and nutrient composition of experimental diets (%, as-fed basis).

Items	Diet^1^		
	TS	CS	PS
Ingredient, %			
Tapioca starch	59.00		
Corn starch		59.00	
Pea starch			59.00
Soybean meal	27.00	27.00	27.00
Corn gluten meal	3.40	3.40	3.40
Wheat bran	3.36	3.36	3.36
Soybean oil	3.25	3.25	3.25
L-Lysine-HCl (98%)	0.30	0.30	0.30
DL-Methionine	0.13	0.13	0.13
L-Threonine	0.06	0.06	0.06
Dicalcium phosphate	1.50	1.50	1.50
Limestone	0.30	0.30	0.30
Choline chloride (50%)	0.40	0.40	0.40
Salt	0.30	0.30	0.30
Vitamin and mineral Premix^2^	1.00	1.00	1.00
Total	100.00	100.00	100.00
Calculated content^3^			
ME^4^, MJ/kg	13.81	13.81	13.81
Standardized ileal digestible amine acid, %			
Lysine	0.88	0.88	0.88
Methionine + Cysteine	0.49	0.49	0.49
Threonine	0.50	0.50	0.50
Tryptophan	0.15	0.15	0.15
Analyzed nutrient composition			
^5^Dry matter, %	88.48	88.86	88.45
^5^Crude protein, %	14.54	14.55	14.55
^5^Crude fat, %	1.26	1.25	1.25
^5^Crude ash, %	4.09	4.09	4.11
Total starch, % DM	52.25	52.26	52.25
Amylose/amylopectin	0.11	0.25	0.44

*^1^TS, tapioca starch; CS, corn starch; PS, pea starch. ^2^Provided per kilogram of complete diet: vitamin A, 15,000 IU; vitamin D3, 3,000 IU; vitamin E, 150 mg; vitamin K3, 3 mg; vitamin B1, 3 mg; vitamin B2, 6 mg; vitamin B6, 5 mg; vitamin B12, 0.03 mg; niacin, 45 mg; vitamin C, 250 mg; calcium pantothenate, 9 mg; folic acid, 1 mg; biotin, 0.3 mg; choline chloride, 500 mg; Fe (FeSO_4_.H_2_O), 170 mg; Cu (CuSO_4_.5H_2_O), 150 mg; I (KI), 0.90 mg; Se (Na_2_SeO_3_),0.2 mg; Zn (ZnSO_4_.H_2_O), 150 mg; Mg (MgO), 68 mg; Mn (MnSO_4_.H_2_O), 80 mg; Co (CoCl_2_), 0.3 mg. ^3^Values were estimated based on database of NRC (2012). ^4^ME, metabolized energy. ^5^Analytical results obtained according to AOAC (2007).*

At the end of the experiment, eight pigs from each group (*n* = 8 barrows, based on the average body weight in each pen) were selected and then sampled. After fasting for approximately 12 h, the pigs were euthanized by electrical stunning and exsanguination. The digesta of the colon was collected and homogenized and the pH values of the digesta was immediately determined. About 5 g of mixed colonic digesta were frozen in liquid nitrogen and then stored at -80°C for later bacterial DNA isolation and metabolites analysis. Another 10 g of mixed colonic digesta were stored at -20°C for starch, amylose, and amylose/amylopectin ratio analyses.

### DNA Extraction, Illumina MiSeq Sequencing, and Data Processing

Total genomic DNA from the individual samples of colonic digesta was extracted using a QIAamp PowerFecal DNA Kit (QIAGEN, Hilden, Germany) according to the manufacturer’s instructions. DNA concentrations of every sample were quantified using a Nanodrop 2000 spectrophotometer (Thermo Fisher Scientific, Wilmington, DE, United States). The genes of all bacterial 16S rRNA in the region of V3–V4 were amplified by polymerase chain reaction (PCR) using a universal forward primer 338F (5′-ACTCCTRCGGGAGGCAGCAG-3′) and a reverse primer 806R (5′-GGACTACCVGGGTATCTAAT-3′) (Mao et al., 2015b). PCR amplicons were purified using the AxyPrep DNA Gel Extraction Kit (Axygen Biosciences, Union City, CA, United States), according to the manufacturer’s instructions. The purified amplicons were pooled in equimolar from each sample and paired-end sequenced (2 × 250) on an Illumina MiSeq platform (Majorbio, Shanghai, China) according to the standard protocols (Caporaso et al., 2012).

The QIIME (version 1.17) software package was used to demultiplex and quality-filter raw sequence data generated from 16S rRNA MiSeq sequencing (Campbell et al., 2010). Gaps in each sequence were discarded from all the samples to decrease the noise generated the screening, filtering, and pre-clustering processes as described previously (Gao et al., 2018). Operational taxonomic units (OTUs) were clustered as a similarity cut-off of 97% using UPARSE (version 7.1^1^) and unnormal gene sequences were identified and deleted using UCHIME (Edgar, 2010). With each OTU, the representative sequence was analyzed using the Ribosomal Database Project (RDP) classifier (RRID: SCR_006633) against the Silva (SSU119) 16S rRNA database employing a confidence level of 90%.

The bacterial diversity, such as rarefaction analysis, the number of observed OTUs, coverage abundance estimator, richness estimator (Chao 1 and ACE), and diversity indices (Shannon and Simpson) were calculated using MOTHUR software (version 1.35.1^2^) according to previous instructions (Schloss et al., 2009). Principal coordinates analysis (PCoA) was performed based on the Bray–Curtis distance, and analysis of molecular variance (AMOVA) was performed to compare the dissimilarities among samples using the MOTHUR (Schloss et al., 2009).

The 16S sequencing data generated in this study were deposited into the National Center of Biotechnology Information (NCBI) Sequence Read Archive (SRA) database under Accession No. PRJNA517450.

### Chemical Composition Analysis

The pH value of the colonic digesta was detected using a protable pH meter (HI 9024C; HANNA Instruments, Woonsocket, RI, United States). The starch and amylose concentrations of diets and colonic digesta were analyzed using the Total Starch Kit and Amylose/Amylopectin Kit (Megazyme International, Wicklow, Ireland), respectively. SCFA concentrations in the colon were determined by gas chromatography (GC) according to the method described in a previous study (Yu et al., 2017). Colonic lactate concentration was measured using a commercial kit according to the manufacturer’s instructions (Nanjing Jiancheng Biological Engineering Institute, Nanjing, China).

Ammonia concentration in the colon was measured using a spectrophotometer (UV-2450; Shimadzu, Tokyo, Japan) according to a previous method (Chaney and Marbach, 1962). The biogenic amines concentrations in the colonic digesta were measured using high-performance liquid chromatography (HPLC) with precolumn dansylation according to a previous study (Yang et al., 2014). The concentrations of phenolic and indolic compounds in the colonic digesta were analyzed by HPLC, as described previously (Schüssler and Nitschke, 1999), with slight modifications. Briefly, 0.1 g of colonic digesta was mixed with 1.0 mL of acetonitrile. The mixture was vortexed, stored at -20°C for 20 min, and then centrifuged at 3000 ×*g* for 10 min at 4°C. The supernatant was filtered through a 0.22-μm membrane and then analyzed on a Waters alliance HPLC System (e2695 separation module: Waters, Milford, MA, United States) with a Multi λ Fluorescence Detector (2475: Waters, Milford, MA, United States). Gradient elution of two mobile phases was used: mobile phase A consisted of HPLC grade water, and mobile phase B was acetonitrile. The gradient program was: 82% A initially, 55% A at 12 min, 10% A at 22 min, and 100% B at 23 min. The flow rate was 1.0 mL/min and the column temperature were 30°C.

### Gas Chromatograph–Time-of-Flight Mass Spectrometry Analysis

GC-TOF/MS was used to measure the colonic metabolites. All of the samples were pretreated, extracted, and identified using the procedure a previously described (Mao et al., 2015a). The LECO Chroma TOF4.3X software, LECO-Fiehn Rtx5 database, and commercial databases, including KEGG^3^ and HMDB^4^, were utilized to extract the raw peak and filter data baseline, as well as further identify and validate the different metabolites. The peaks area of each metabolite was standardized using internal standard normalization methods before further analysis. The resulting data containing the peak number, sample name, and normalized peak were imported into SIMCA-P 13.0 (Umetrics, Umeå, Sweden) for partial least squares discriminant analysis (PLS-DA) and orthogonal partial least-squared discriminant analysis (OPLS-DA). In the present study, the discriminated metabolites were selected based on variable importance in the projection (VIP) value from the OPLS-DA model. VIP > 1 and *q* < 0.05 [false discovery rate (FDR)] were used to select the significant metabolites among the three dietary treatment groups.

### Data Analysis

Statistical calculations for all the experimental data were conducted using the SPSS software package (SPSS v. 20.0: SPSS, Chicago, IL, United States). Before assessing the differences between the groups, the Shapiro–Wilk test was used to confirm whether the variables exhibited a normal distribution. The variables that showed a non-normal distribution (some data of taxa richness and metabolomics parameters) were analyzed by Kruskal–Wallis one-way analysis of variance (ANOVA) with the Benjamini and Hochberg false discovery rate (FDR) multiple-testing correction (Benjamini and Hochberg, 1995). The variables that showed a normal distribution (pH, and metabolite concentrations) were analyzed by one-way ANOVA with a Tukey *post hoc* test. Significant differences were declared at *P* ≤ 0.05. The correlation between significantly changed bacteria by diet types (at the genus level, adjusted *P* < 0.05) and pH values, metabolites (VIP > 1.5, adjusted *P* < 0.05, and similarity > 600), main SCFA, and amines were analyzed by Spearman’s rank correlation test using GraphPad Prim version 5.0 (GraphPad Software, San Diego, CA, United States). To elucidate potential systemic properties, we focused on the absolute Spearman’s correlation coefficient > 0.5 with statistical significance at *P* < 0.05. These correlation networks were visualized using Cytoscape 3.5.1 software (Smoot et al., 2011).

## Results

### Growth Performance, the Content of Starch, Amylose, Amylose/Amylopectin Ratio of Colonic Digesta

In this study, the pigs in the PS group showed a greater BW and ADG than those in the TS group (BW: 115.88 ± 1.21 vs. 109.52 ± 0.91 kg/d; ADG: 0.97 ± 0.03 vs. 0.85 ± 0.01 kg/d) and a lower F:G than those in TS group (3.01 ± 0.04 vs. 3.34 ± 0.09 kg/d) during the whole experimental period (*P* < 0.05). However, there was no difference in ADFI (*P* = 0.741) among the TS group (2.83 ± 0.07), CS group (2.85 ± 0.05), and PS group (2.89 ± 0.05).

As shown in Supplementary Table S1, pigs in the PS group showed a higher starch and amylose/amylopectin ratios in colonic digesta than those in the TS group (*P* < 0.05). The starch content of the CS group in colonic digesta was lower than that of the PS group, but higher than that of the TS group. Additionally, there was no significant difference on the content of amylose in colonic digesta among the three groups.

### Colonic Bacterial Community Structure

**FIGURE 1:**
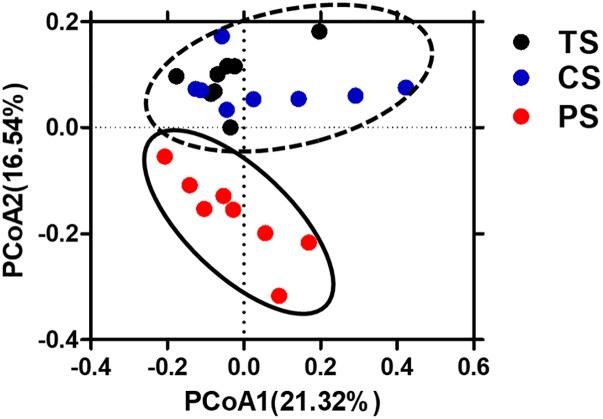
Principal coordinates analysis (PCoA) of bacterial communities in the colonic digesta of pigs (based on the Bray–Curtis distance). Circles with dash line or solid indicate that groups were significantly distinct using AMOVA analysis (*P* < 0.05). TS, tapioca starch; CS, corn starch; PS, pea starch.

To evaluate the impact of the different starch diets on the microbial composition of colonic digesta, a total of 998,521 V3–V4 16S rRNA effective sequences from the 24 samples, with an average of 41,605 sequences per sample were used for subsequent analysis. The flattened rarefaction curves showed that the sampling in each group provided sufficient OTU coverage (Supplementary Figure S1). The richness and diversity of the colonic digesta bacteria are shown in Table 2. Pigs in the CS group had a higher species richness and diversity indices compared to that in the PS group, as reflected by the OTU numbers, Chao1, and Shannon index with statistical differences. However, the ACE richness index, coverage, and Simpson index did not differ among the different groups. The PCoA with Bray–Curtis distance results showed that the PS group separated from the TS and CS groups (Figure 1). AMOVA analysis also showed significant dissimilarities among the three groups (*Fs* = 2.84, *P* < 0.001, among TS, CS, and PS groups; *Fs* = 3.31, *P* < 0.001, TS vs. PS; *Fs* = 3.45, *P* < 0.001, CS vs. PS; *Fs* = 1.80, *P* < 0.05, TS vs. CS).

**Table 2:** Summary statistic of colonic digesta bacterial community at the 3% dissimilarity level.

Items	Treatment			*P*-value
	TS	CS	PS	
OTU numbers	857 ± 13^ab^	901 ± 15^a^	839 ± 17^b^	0.019
Coverage, %	99.51 ± 0.04	99.60 ± 0.03	99.61 ± 0.02	0.112
Richness				
Chao1	995.89 ± 17.56^ab^	1049.74 ± 17.62^a^	977.47 ± 17.62^b^	0.028
ACE	992.51 ± 13.53	1030.63 ± 18.08	975.93 ± 18.36	0.103
Diversity indices				
Shannon	4.67 ± 0.07^ab^	4.87 ± 0.06^a^	4.52 ± 0.09^b^	0.009
Simpson	0.69 ± 0.01	0.72 ± 0.02	0.67 ± 0.02	0.137

*Values are means ± SEM (n = 8). Results were analyzed by one-way analysis of variance (ANOVA) with Turkey’s test, and the variant letter in the same row indicated significant difference when *P* < 0.05. OTU, operational taxonomic units; ACE, abundance-based coverage estimator. TS, tapioca starch; CS, corn starch; PS, pea starch.*

**FIGURE 2:**
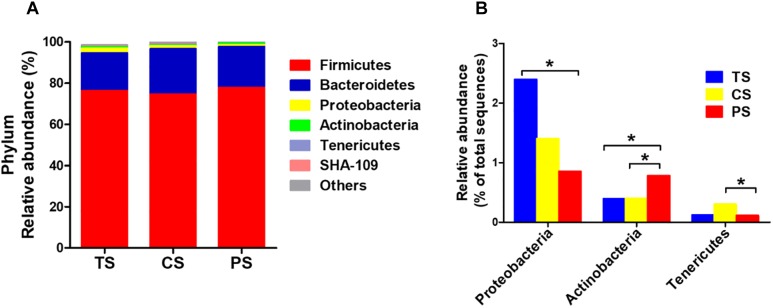
Relative abundance of bacterial phylum in the colonic digesta of pigs **(A)**. The significantly changed phyla in the colonic digesta **(B)**. The values were expressed as the medians (*n* = 8). Statistical differences were calculated by Kruskal–Wallis *H*-test: ^∗^*P* < 0.05. TS, tapioca starch; CS, corn starch; PS, pea starch.

At the phylum level, the Firmicutes and Bacteroidetes were the two predominant phyla, contributing 77.79 and 17.59% in the TS group, 74.17 and 20.97% in the CS group, and 78.05 and 18.96% in the PS group, respectively (Figure 2A). Proteobacteria and Actinobacteria were the next two most dominant phyla, accounting for 3.04 and 0.38% in the TS group, 1.59 and 0.50% in CS group, and 1.01 and 0.99% in the PS group, respectively. The abundance of the phyla Proteobacteria in the PS group was significant decreased (*P* < 0.05) compared with that in the TS group. There was a higher abundance of Tenericutes in the PS group than that in CS group (*P* < 0.05, Figure 2B). In addition, the abundance of Actinobacteria in the PS group was increased (*P* < 0.05) compared with that in the TS and CS groups. However, no significant changes were found in the abundance of Firmicutes, Bacteroidetes, and SHA-109 among the three groups.

**FIGURE 3:**
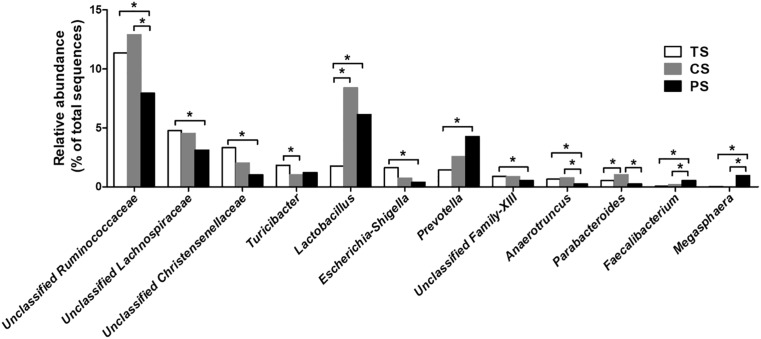
Significantly changed bacteria genera by different starch source diet treatment. The values were expressed as the medians (*n* = 8). Statistical differences were calculated by Kruskal–Wallis test: ^∗^FDR-adjusted *P*-value < 0.05. TS, tapioca starch; CS, corn starch; PS, pea starch.

At the genus level, the 30 most dominating genera of the colonic digesta are presented in a heat map (Supplementary Figure S2). The eight most dominating genera (those with a relative abundance ≥ 5% in at least one treatment) were the *Clostridium*_sensu_stricto_1, unclassified Ruminococcaceae, unclassified Peptostreptococcaceae, unclassified S24-7, *Lactobacillus*, *Streptococcus*, unclassified Lachnospiraceae, and *Prevotella*. The pigs in the PS group showed a lower relative abundance of unclassified Ruminococcaceae, unclassified Lachnospiraceae, unclassified Christensenellaceae, *Escherichia–Shigella*, unclassified Family-XIII, and *Anaerotruncus* compared with those in the TS group (adjusted *P* < 0.05), while the relative abundance of *Lactobacillus*, *Prevotella*, *Faecalibacterium*, and *Megasphaera* were higher (adjusted *P* < 0.05) (Figure 3). Meanwhile, the pigs in the PS group had a lower relative abundance of unclassified Ruminococcaceae, *Anaerotruncus*, and *Parabacteroides* (adjusted *P* < 0.05) compared with the pigs in the CS group, while had a higher relative abundance of *Faecalibacterium* and *Megasphaera* (adjusted *P* < 0.05). In addition, the abundance of *Lactobacillus* and *Parabacteroides* were also increased in the CS group compared with the TS group (adjusted *P* < 0.05).

### Metabolite Profiles in the Colonic Digesta

**FIGURE 4:**
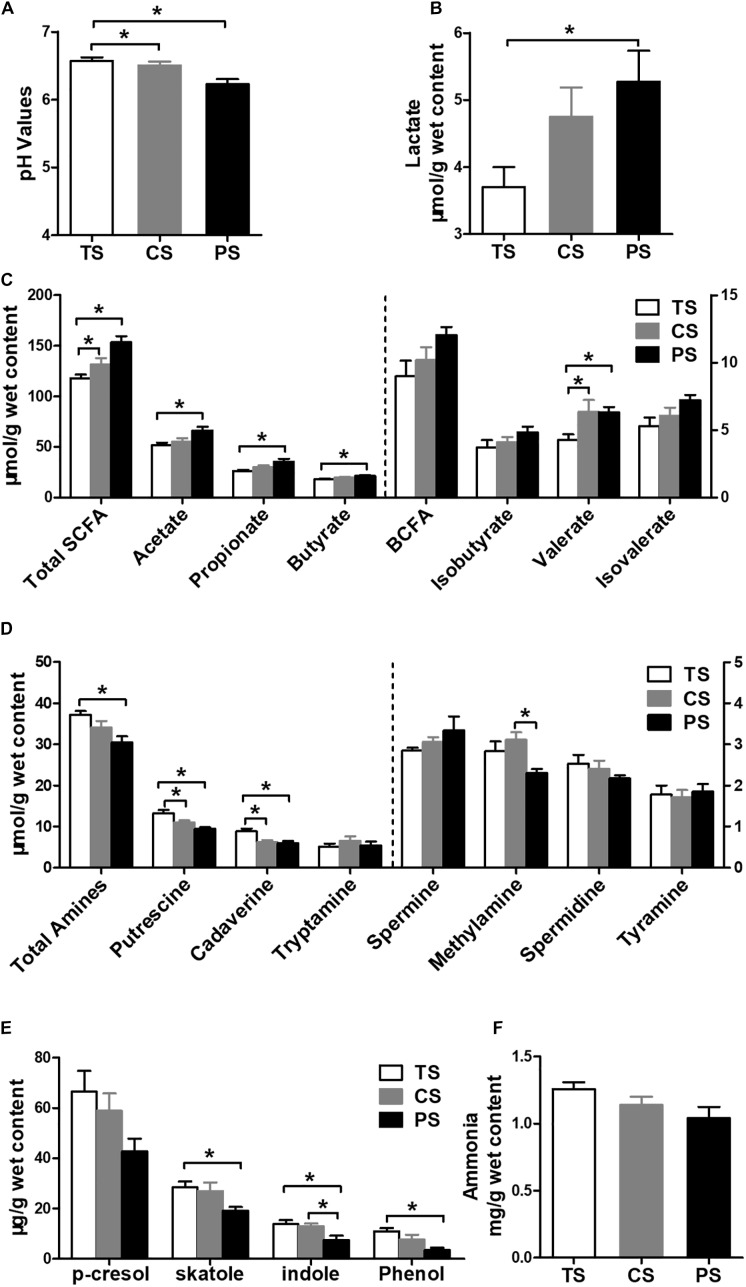
Effects of different starch source diet on the pH values and microbial metabolites in colonic digesta of pigs. **(A)** pH values; **(B)** lactate; **(C)** SCFAs; **(D)** biogenic amines; **(E)** phenolic and indole compounds; **(F)** ammonia. The values were expressed as the means ± SEM (*n* = 8). Asterisks indicated statistically significant difference among different treatment groups: ^∗^*P* < 0.05. TS, tapioca starch; CS, corn starch; PS, pea starch.

As shown in Figure 4A, pigs in the CS and PS groups presented with significantly decreased pH values compared with the TS group (*P* < 0.05). Pigs in the PS group had a higher lactate concentration than it in the TS group (*P* < 0.05) (Figure 4B). For SCFA (Figure 4C), the concentrations of total SCFA, acetate, propionate, butyrate, and valerate were higher in the PS group compared with the TS group (*P* < 0.05). Meanwhile, the concentrations of total SCFA and valerate were also increased in the CS group compared with the TS group (*P* < 0.05). However, the concentrations of branched-chain fatty acid (BCFA), isobutyrate, and isovalerate were not affected by the dietary treatments (*P* > 0.05).

For biogenic amines (Figure 4D), the pigs in the PS group had a lower total amines, putrescine, and cadaverine concentrations than in the TS group (*P* < 0.05), and a lower methylamine concentration than in the CS group (*P* < 0.05). The pigs in the CS group also had lower putrescine and cadaverine concentrations than in the TS group (*P* < 0.05). However, there were no differences of tryptamine, spermine, spermidine, or tyramine concentrations among different dietary treatments (*P* > 0.05). For phenolic and indole compounds (Figure 4E), the concentrations of skatole, indole, and phenol were lower in the PS group than those in the TS group (*P* < 0.05). The concentration of indole was also lower in the CS group than in the TS group (*P* < 0.05). The concentration of *p*-cresol was not affected by the dietary treatments (*P* > 0.05). The dietary treatments also did not affect the ammonia concentration (*P* > 0.05; Figure 4F).

**FIGURE 5:**
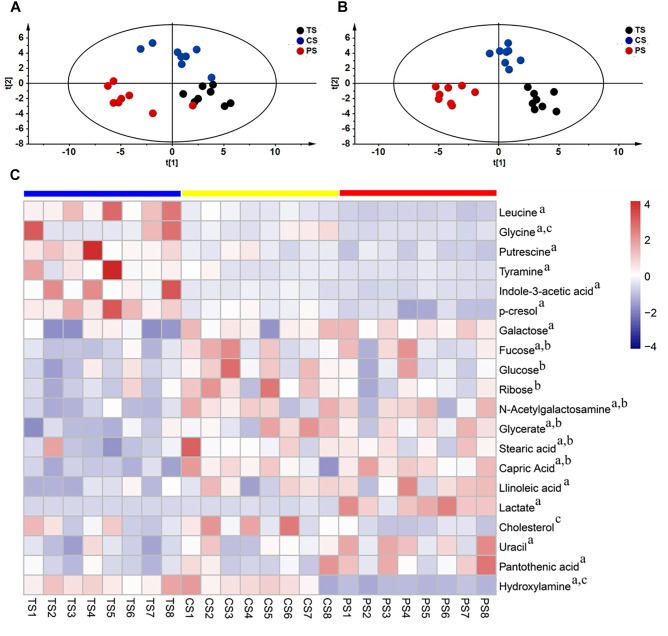
The colon metabolome of pigs. Partial least squares discriminant (PLS-DA) **(A)** and orthogonal partial least-squares discriminant (OPLS-DA) **(B)** scores of metabolomics comparison in pigs fed with different starch source diet. **(C)** Heat-map visualizing the significantly changed metabolites. Metabolites peak area were Z score transformed. Blue *squares* tapioca starch diet (TS), Yellow *squares* corn starch diet (CS), Red *squares* pea starch diet (PS). a, b, c indicated statistically significant difference between TS and PS, between TS and CS, between CS and PS, respectively.

To further predict whether the feeding different starch diets affected the metabolite profiles of the colonic digesta, GC-TOF/MS was used to analyze the metabolite profiles. A total of 689 valid peaks were detected, and 135 reliable metabolite compounds were quantified in all the samples (Similarity > 600), and these mainly included amino acids, amines, fatty acids, carbohydrates, organic acids, purines, lipids, and others. The PLS-DA (Figure 5A) and OPLS-DA (Figure 5B) models showed that the three groups were well-separated.

To assess which compounds were responsible for the differences among the three groups, the parameters of VIP > 1.0 and adjusted *P* < 0.05 were used as key lineages for separating the colonic compounds among the three groups (Figure 5C and Supplementary Table S2). In total, twenty compounds with a VIP > 1.0 and adjusted *P* < 0.05 were identified. Among these, seven metabolites (leucine, glycine, putrescine, tyramine, indole-3-acetic acid, *p*-cresol, and hydroxylamine) were reduced and 10 metabolites (galactose, fucose, *N*-acetylgalactosamine, glycerate, stearic acid, capric acid, linoleic acid, lactate, uracil, and pantothenic acid) were enriched in the pigs fed the PS diet compared with the TS diet. Meanwhile, three metabolites (glycine, cholesterol, and hydroxylamine) were reduced in the pigs fed with the PS diet compared with the CS diet. Additionally, seven metabolites (fucose, glucose, ribose, *N*-acetylgalactosamine, glycerate, stearic acid, and capric acid) were enriched in the pigs fed with the CS diet compared with the TS diet. Overall, these results indicated that the PS diet (containing a high ratio of amylose) markedly increased the concentrations of organic acids (acetate, propionate, butyrate, valerate, lactate), carbohydrates, and lipids related compounds, and decreased the concentrations of amino acid related compounds (leucine, glycine, amines, phenol, and indole compounds), suggesting a strong impact of the PS diet on carbohydrate, lipid, and amino acid metabolism characteristics in the colon.

### Correlation Analysis Between the Colonic Metabolome and Microbiome

**FIGURE 6:**
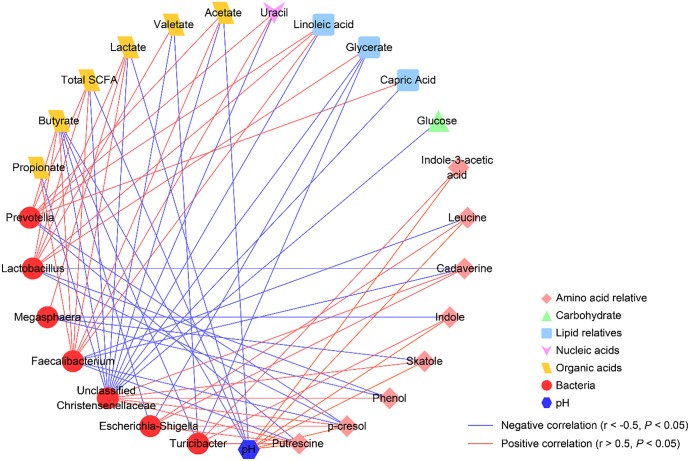
Correlation network analysis among the microbiota (at the genera level) affected by different starch diets treatment and the potential marker compounds (VIP > 1.5, *q* < 0.05, similarity > 600). Each line among predominant bacteria (relative abundance) at the genus level and metabolites (peak areas or concentrations) has an absolute Spearman rank correlation above 0.50 [*blue lines*, negative correlation (*r* ≤ –0.50); *red lines*, positive correlation (*r* ≥ 0.50)] with a *P* < 0.05 are presented.

To explore the functional correlation between changes in the colonic microbiome and metabolite profiles, a Spearman’s rank correlation analysis matrix was generated by calculating the Spearman’s correlation coefficient among the microbial composition affected by the diet treatments (at the genus level, adjusted *P* < 0.05), pH values, and metabolites (Figure 6). A clear significant correlation (*P* < 0.05) and an absolute value of the Spearman’s correlation coefficient of *r* > 0.5 was identified between the changes in the colonic microbiome and the metabolome. The correlation analysis revealed that *Lactobacillus* was positively correlated with glycerate, linoleic acid, total SCFA, butyrate, valerate, and lactate (*P* < 0.05), while negatively correlated with cadaverine, *p*-cresol, and phenol (*P* < 0.05). *Prevotella* was positively correlated with capric acid, linoleic acid, uracil, total SCFA, acetate, and butyrate (*P* < 0.05), while was negatively correlated with putrescine and *p*-cresol (*P* < 0.05). Unclassified Christensenellaceae was positively correlated with putrescine, cadaverine, *p*-cresol, skatole, and phenol (*P* < 0.05), while was negatively correlated with glucose, glycerate, capric acid, linoleic acid, uracil, total SCFA, acetate, propionate, butyrate, valerate, and lactate (*P* < 0.05). *Turicibacter* was positively correlated with putrescine, indole-3-acetic acid, and indole (*P* < 0.05), while was negatively correlated with glycerate, butyrate, and valerate (*P* < 0.05). *Escherichia–Shigella* was positively correlated with leucine, putrescine, cadaverine, and *p*-cresol (*P* < 0.05), while was negatively correlated with glycerate and butyrate (*P* < 0.05). *Megasphaera* was positively correlated with lactate (*P* < 0.05), while was negatively correlated with skatole, indole, and phenol (*P* < 0.05). *Faecalibacterium* was negatively correlated with leucine, putrescine, cadaverine, and *p*-cresol (*P* < 0.05), while was positively correlated with linoleic acid, uracil, total SCFA, acetate, propionate, butyrate, and lactate (*P* < 0.05). Meanwhile, our results also revealed that colonic pH was positively correlated with leucine, putrescine, indole-3-acetic acid, *p*-cresol, skatole, and indole (*P* < 0.05), while negatively correlated with total SCFA, acetate, butyrate, and lactate (*P* < 0.05). Collectively, these results indicated that the changes in the colonic digesta microbiota were correlated with alterations of metabolites in pigs.

## Discussion

Starch is the main dietary energy source for humans and monogastric animals, and previous studies have indicated a close relationship between the structure of dietary starch types and their utilization efficiency (Pieper et al., 2008; Jha et al., 2011). However, there is a paucity of information on the microbial community and the metabolic profile after treatment with different starch sources. In the present study, we investigated the response of the microbes and metabolites of colonic digesta of pigs fed different starch sources using 16S rRNA MiSeq sequencing, GC-TOF/MS, and biochemical analyses. Our results showed that treatment with different dietary starch led to different responses regarding microbial composition and metabolism in the colon. The PS diet (containing high ratio of amylose) markedly increased the abundance of some probiotics (such as *Lactobacillus*), while decreased the abundance of *Escherichia coli* compared with the TS diet (containing a low ratio of amylose). Moreover, our results also demonstrated that the PS diet increased the concentrations of organic acids (SCFAs and lactate) and some macronutrients (galactose, fucose, glucose, ribose, stearic acid, and linoleic acid) compared with the TS diet, and decreased the amino acids and their derivatives (leucine, glycine, amines, phenolic and indole compounds). These findings indicated a marked influence of the different dietary starch sources on the intestinal microbial community and metabolic profiles in the colon of pigs.

### Diets With Different Starch Sources Altered the Colonic Microbiota Structure in Pigs

Substrate availability and the preferential substrate utilization of microbes are the major factors that affects the composition of gastrointestinal tract microbiota (Castillo et al., 2007). In the present study, we found that the PS diet resulted in a lower pH and reduced the bacterial richness and diversity, as indicated by the Chao 1 values and Shannon index. Moreover, the results of Bray–Curtis PCoA and AMOVA analyses further revealed difference in the colonic bacterial communities among the three groups. Many amylolytic bacteria can produce bacteriocins and antimicrobial molecules and then prevent the colonization of bacteria that cannot utilize starch (Harlow et al., 2016). Thus, one potential explanation for altered the colonic bacterial richness and diversity may be due to the PS diet increased the abundance of some amylolytic bacteria, such as *Lactobacillus*, *Prevotella*, *Faecalibacterium*, and *Megasphaera* as mentioned below, and then inhibit the colonization of many bacteria that cannot utilize starch. Bacteria-specific factors, such as substrate affinity, substrate preference, and pH tolerance, could influence competition among amylolytic bacteria. A lower pH value in the colon might decrease the richness of some bacteria (such as *Escherichia–Shigella*) due to their susceptibility to low pH and this can also increase the abundance of several low-pH-tolerant colonic digesta bacteria (Daniëlle et al., 2013; Mao et al., 2015a). Therefore, other potential explanation for the decreased colonic bacterial richness and diversity in the pigs fed the PS diet may be due to the low pH. Furthermore, the digestion of TS allows for fast digestion (Weurding et al., 2001), CS is partially protected by the endosperm protein matrix (Svihus et al., 2005), and PS has a high amylose proportion and cell structures enclosing the starch granules, making it resistance to α-amylase digestion in the small intestine to a certain extent (Sun et al., 2006). Therefore, the alteration in the structure of the microbial population may also be due to the PS diet resulting in some amount of fermentable substrate (starch) entering the colon, thus promoting the growth of amylolytic and other starch-digesting bacterial species.

At the genus level, univariate statistical analysis indicated that the PS diet marked increased the abundance of *Lactobacillus*, *Prevotella*, *Faecalibacterium*, and *Megasphaera* in the colonic digesta compared with the TS diet. Similarly, previous studies also demonstrated that diets rich in amylose increased the abundance of *Lactobacillus* (Bird et al., 2007; Luo et al., 2015; Newman et al., 2018), *Prevotella* (Sun et al., 2015; Maier et al., 2017), *Faecalibacterium* (Daniëlle et al., 2013; Wang et al., 2018), and *Megasphaera* (Newman et al., 2018) in both humans and pigs. Among these various taxa, several species of *Lactobacillus* have many beneficial effects on the gut health of both humans and animals (Azad et al., 2018; Yu et al., 2018), normalizing the ratio of anti-inflammatory to pro-inflammatory cytokines and inhibiting the infection or colonization of pathogens via the productions of antimicrobial factors, such as bacteriocins and lactate (O’Mahony et al., 2005; Yu et al., 2018). *Prevotella* is well-known as a gut colonizer, one of the predominant starch-degrading bacteria in the intestine, and confirming the producing capacity of SCFAs (Flint et al., 2012). *Faecalibacterium* and *Megasphaera* are starch-utilizing commensal bacteria that can ferment starch to produce butyrate (Harry et al., 2008; Kamke et al., 2016). Some species in the genus *Faecalibacterium* and *Megasphaera* together with butyrate, have many beneficial effects in regard to colonic homeostasis via enhancement of epithelial energy metabolism and stimulating immune system balance (Sokol et al., 2008; Petra et al., 2014). Thus, the higher relative abundances of some beneficial bacteria (*Lactobacillus*, *Prevotella*, *Faecalibacterium*, and *Megasphaera*) in the PS group indicated that feeding of PS diet (rich in amylose) might have beneficial effects on the colonic health of pigs.

Additionally, pigs feed the PS diet demonstrated a decrease in the abundance of unclassified Ruminococcaceae, unclassified Lachnospiraceae, unclassified Christensenellaceae, *Escherichia–Shigella*, and *Anaerotruncus* compared with the TS diet. Previous study also indicated that a high amylose diet decreased several of above bacterias in the colon (Bird et al., 2007; Newman et al., 2018). Ruminococcaceae and Lachnospiraceae are the main families in the gut of mammals and have been associated with the maintenance of gut health (Donaldson et al., 2016). Previous studies have found that the enrichment of these families is associated with colonic mucosal inflammation, which can trigger colitis upon disruption of the barrier function of colonic epithelial cell (Willing et al., 2011; Nakanishi et al., 2015). *Escherichia–Shigella* is involved in protein utilization and is sensitive to acidic environment (Chen et al., 2018). Thus, the decrease in *Escherichia–Shigella* may be explained by the shortage of protein substrates for fermentation and the lower pH after feeding with the PS diet feeding. Some species within the genus *Escherichia–Shigella*, such as *E. coli*, are known as opportunistic pathogens and are associated with numerous infections and diseases, such as bacillary dysentery or colitis disease (Barnich et al., 2007). The enrichment of some species of *Anaerotruncus*, which belongs to *Clostridium* cluster IV, are associated with inflammatory bowel disease in the feces and rectal mucosa of humans (Satokari et al., 2014). Therefore, these findings suggested that feeding of a PS diet (rich in amylose) inhibited the abundance of several potential pathogens, and this may also have beneficial effects on the health of pigs.

### Diets With Different Starch Sources Significantly Altered the Colonic Metabolite Profiles of Pigs

In the intestine, differences in substrate fermentation by microbiota also lead to different microbial metabolic process and metabolite profiles (Fouhse et al., 2015). In our study, PLS-DA and OPLS-DA analyses showed a clear separation of colonic metabolites due to the different starch diets, indicating significant differences in the metabolic profiles. The univariate statistical analysis indicated that the carbohydrates, such as galactose, fucose, glucose, ribose, and *N*-acetylgalactosamine were increased in the CS and PS groups compared with the TS group, indicating that carbohydrate metabolism was influenced at the local level (Figure 5C and Supplementary Table S2). A previous study also demonstrated that a raw potato starch diet (rich in amylose) could also increase the concentrations of fructose, glucose, and maltose (Sun et al., 2016). Diets containing starch with a higher content of amylose can decrease the digestibility of starch in the small intestine and lead to most of the starch being extensively passed into the hindgut (Regmi et al., 2011), where it can be fermented by microbes to produce sugars. Indeed, our study also found that the colonic starch content in the pigs fed with the CS and PS diets were significantly higher than that in the pigs fed with the TS diet (Supplementary Table S1). Thus, these changes in sugar concentrations may be deemed a fundamental alteration caused by the CS and PS diets rich in amylose. Meanwhile, several fatty acids, such as stearic acid, capric acid, and linoleic acid, were also increased in the colon of the PS group when compared with the TS group. Resistant starch (with a high ratio of amylose) can regulate lipid metabolism and decrease the absorption of fatty acids (Lee et al., 2012), which may partly explain this observation.

Our study also showed that the PS diet significantly increased the concentrations of organic acids, such as total SCFAs, acetate, propionate, butyrate, and lactate. Similarly, gut SCFAs and lactate concentrations increased when pigs are fed with high amounts of amylose (Topping et al., 1997; Bird et al., 2007; Fouhse et al., 2015) and this shift in metabolites may be attributed to increase in SCFA- and lactate-producing bacteria. The correlation analysis also showed a positive correlation between these metabolites and the abundance of *Prevotella*, *Faecalibacterium*, *Megasphaera*, and *Lactobacillus*. SCFAs and lactate have a beneficial role in the metabolic functions and health of the gut. Acetate and propionate are the energy substrates of peripheral tissues, butyrate is the major energy source for colonic epithelial cells and exerts an anti-inflammatory function, and lactate can inhibit the activity of pathogens that invade the gut, such as *Escherichia–Shigella* (Tremaroli and Bäckhed, 2012). Therefore, the increase in SCFAs and lactate concentrations in the present study suggest the presence of a host-friendly gut environment after feeding of the PS diet.

Additionally, results of colonic metabolomics and biochemical analyses showed that the PS diet feeding significantly decreased the amino acid relatives compared with the TS group, such as leucine and glycine, indicating fewer nitrogen sources left for the microbial fermentation. This alteration may be due to the PS diet which increased the amount of fermentable substrate (starch) entering the colon and the carbon: nitrogen ratio of the substrates for microbial fermentation. Furthermore, ammonia, several amines, as well as phenolic and indole compounds were also decreased in the pigs fed with the PS diet compared to those in the pigs fed with the TS diet. Biogenic amines are formed from decarboxylation of amino acids by gut bacteria, and phenolic and indole compounds are produced from decarboxylation of aromatic amino acids by gut bacteria, such as *Escherichia–Shigella* (Blachier et al., 2007). Correspondingly, our results also showed that the PS diet markedly decreased the abundance of *Escherichia–Shigella*, and this could explain the lower levels of amines as well as phenolic and indole compounds. On the other hand, amino acid fermentation is favored at a neutral pH, as the proteases secreted by the bacteria are more active at a neutral or slightly alkaline pH than an acidic pH (Pi et al., 2018). In the current study, the pH in the colonic digesta was maintained at a more acidic level in the PS group compared with that in the TS group (mean of 6.21 and 6.61 in the PS and TS group, respectively; Figure 4A). Thus, the lower pH in the PS group may influence the protease activity, which may further support the conclusion that the lower concentrations of amines, as well as phenolic and indole compounds in the PS group originated from changes in the microbial proteolytic activity. High level of amines (such as cadaverine and putrescine), phenol, and skatole might be toxic to gut health (Davila-Gay et al., 2013; Yin et al., 2018b). Thus, decreasing the concentrations of these compounds via a PS diet may exert a beneficial effect on gut health. In general, our findings clearly indicate an evident change in microbial metabolic activity, higher microbial carbohydrate fermentation and lower microbial catabolism of amino acids after feeding of a PS diet.

## Conclusion

The present study combining microbiome, metabolome, and biochemical analyses demonstrated that a PS diet (with greater amylose content) selectively altered the gut microbial composition and metabolic profiles in the colon of the pigs, likely toward a more host-friendly gut environment. Colonic bacteria, such as *Lactobacillus* and many SCFAs-producing bacteria increased, whereas the abundance of *Escherichia–Shigella* decreased after feeding a PS diet. The intestinal metabolites were changed by the different dietary starch sources, as evidenced by the increase in the concentrations of organic acids and carbohydrates and the decrease in the concentrations of metabolites involved in amino-acid metabolism. These findings may help us to understand the effects of dietary starches with higher amylose/amylopection ratios on the nutrition and health of animals and humans.

## Data Availability

All datasets for this study are included in the manuscript and/or the Supplementary Material.

## Author Contributions

MY, WC, and XM conceived and designed the whole trial. ZL and TR conducted the pig trial. MY and ZL conducted laboratory analyses. MY, XM, and GW wrote the manuscript.

## Conflict of Interest Statement

The authors declare that the research was conducted in the absence of any commercial or financial relationships that could be construed as a potential conflict of interest.

## Footnotes

1 http://drive5.com/uparse/

2 http://www.mothur.org

3 http://www.genome.jp/kegg

4 http://www.hmdb.ca
