# Supplementary material for: Microbiome-Metabolomics Analysis Investigating the Impacts of Dietary Starch Types on the Composition and Metabolism of Colonic Microbiota in Finishing Pigs
Source: Front Microbiol. 2019 May 29;10:1143. doi: 10.3389/fmicb.2019.01143 (PMC6549541; doi:10.3389/fmicb.2019.01143)

**Microbiome-metabolomics analysis investigating the impacts of dietary starch types on the composition and metabolism of colonic microbiota in finishing pigs**

Miao Yu, Zhenming Li, Weidong Chen, Ting Rong, Gang Wang, and Xianyong Ma^†^

Institute of Animal Science, Guangdong Academy of Agricultural Sciences; State Key Laboratory of Livestock and Poultry Breeding; Key Laboratory of Animal Nutrition and Feed Science in South China, Ministry of Agriculture; Guangdong Public Laboratory of Animal Breeding and Nutrition; Guangdong Engineering Technology Research Center of animal Meat quality and Safety Control and Evaluation, Guangzhou, China

**Supplementary material**

**Summary**

The supporting information includes 2 supplementary table and 2 supplementary figures.

**Table S1 Content of amylose, amylose/amylopectin ratio of colonic digesta**

| Group | Starch (%) | Amylose (%) | Amylose/amylopectin ratio |
| --- | --- | --- | --- |
| TS | 3.03± 0.12^c^ | 53.29 ± 4.60 | 1.35 ± 0.19^b^ |
| CS | 4.38± 0.42^b^ | 58.34 ± 5.68 | 1.58 ± 0.24^ab^ |
| PS | 8.86± 1.56^a^ | 67.78 ± 7.30 | 1.89 ± 0.17^a^ |
| *P* value | < 0.01 | 0.587 | 0.046 |

Values are means ± SEM (n = 8). Results were analyzed by one-way analysis of variance (ANOVA) with Turkey’s test, and the variant letter in the same row indicated significant difference when *p* < 0.05. TS: tapioca starch; CS: corn starch; PS: pea starch.

**Table S2** Significantly altered metabolites in colonic digesta of pigs

| Metabolites | Biological role | Metabolic pathway | FDR *P* | VIP | The changes in pigs fed higher amylose/amylopectin diet |
| --- | --- | --- | --- | --- | --- |
| Leucine | Amino acid | BCAA metabolism | <0.001 | 1.92 |  |
| Glycine | Amino acid | Glycine and serine metabolism | 0.003 | 1.16 |  |
| Putrescine | Amine | Arginine and proline metabolism | <0.001 | 1.60 |  |
| Tyramine | Amine | Tyrosine metabolism | 0.013 | 1.38 |  |
| Indole-3-acetic acid | Indole and derivatives | Tryptophan metabolism | <0.001 | 1.68 |  |
| *p*-Cresol | Phenols | Tyrosine metabolism | <0.001 | 1.91 |  |
| Galactose | Carbohydrate | Galactose metabolism | 0.020 | 1.46 |  |
| Fucose | Carbohydrate | Fructose and Mannose Metabolism | 0.037 | 1.41 |  |
| Glucose | Carbohydrate | Glycolysis and gluconeogenesis | 0.039 | 1.62 |  |
| Ribose | Carbohydrate | Pentose phosphate pathway | 0.045 | 1.19 |  |
| N-Acetylgalactosamine | Carbohydrate | Others | 0.024 | 1.60 |  |
| Glycerate | Sugar acids and derivatives | Glycerolipid metabolism | 0.019 | 1.62 |  |
| Stearic acid | Long-chain fatty acids | Fatty acid biosynthesis | 0.043 | 1.16 |  |
| Capric acid | Medium-chain fatty acids | Fatty acid biosynthesis | 0.005 | 1.71 |  |
| Linoleic acid | Long-chain fatty acids | Linolenic acid/linoleic acid metabolism | 0.043 | 2.02 |  |
| Lactate | Organic acids | Glycolysis and gluconeogenesis | <0.001 | 2.22 |  |
| Cholesterol | Cholestane derivatives | Sterol synthesized | 0.022 | 1.62 |  |
| Uracil | Pyrimidine | Pyrimidine metabolism | 0.020 | 1.42 |  |
| Pantothenic acid | Cofactors/Vitamins | Pantothenate and CoA biosynthesis | 0.044 | 1.20 |  |
| Hydroxylamine | Inorganic compounds | Others | 0.005 | 1.25 |  |

VIP value was obtained from PLS-DA model with a threshold of 1.0

**Fig. S1** Rarefaction curves comparing the number of sequences with the number of phylotypes found in the 16S rRNA gene libraries from the microbiota in the digesta of the colon of pigs. TS: tapioca starch; CS: corn starch; PS: pea starch.


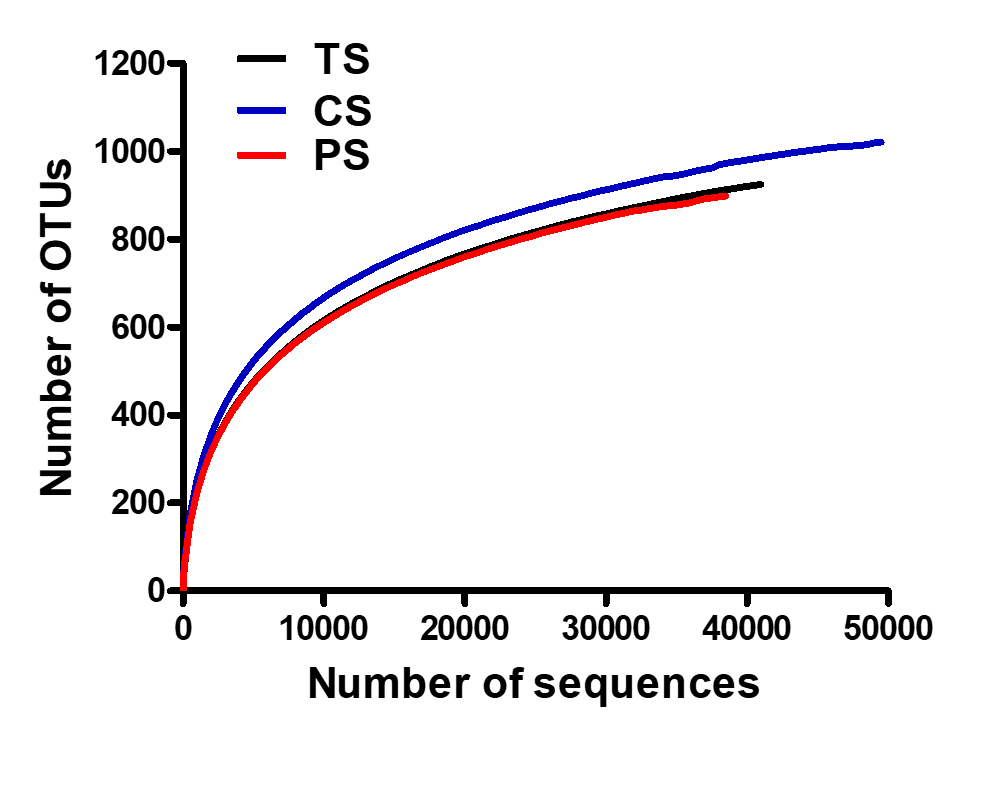


**Fig. S2** Influence of different amylose/amylopectin ratio diet on the 30 most abundant genera in the colonic digesta. The color represents the relative abundance of bacteria. TS: tapioca starch; CS: corn starch; PS: pea starch.


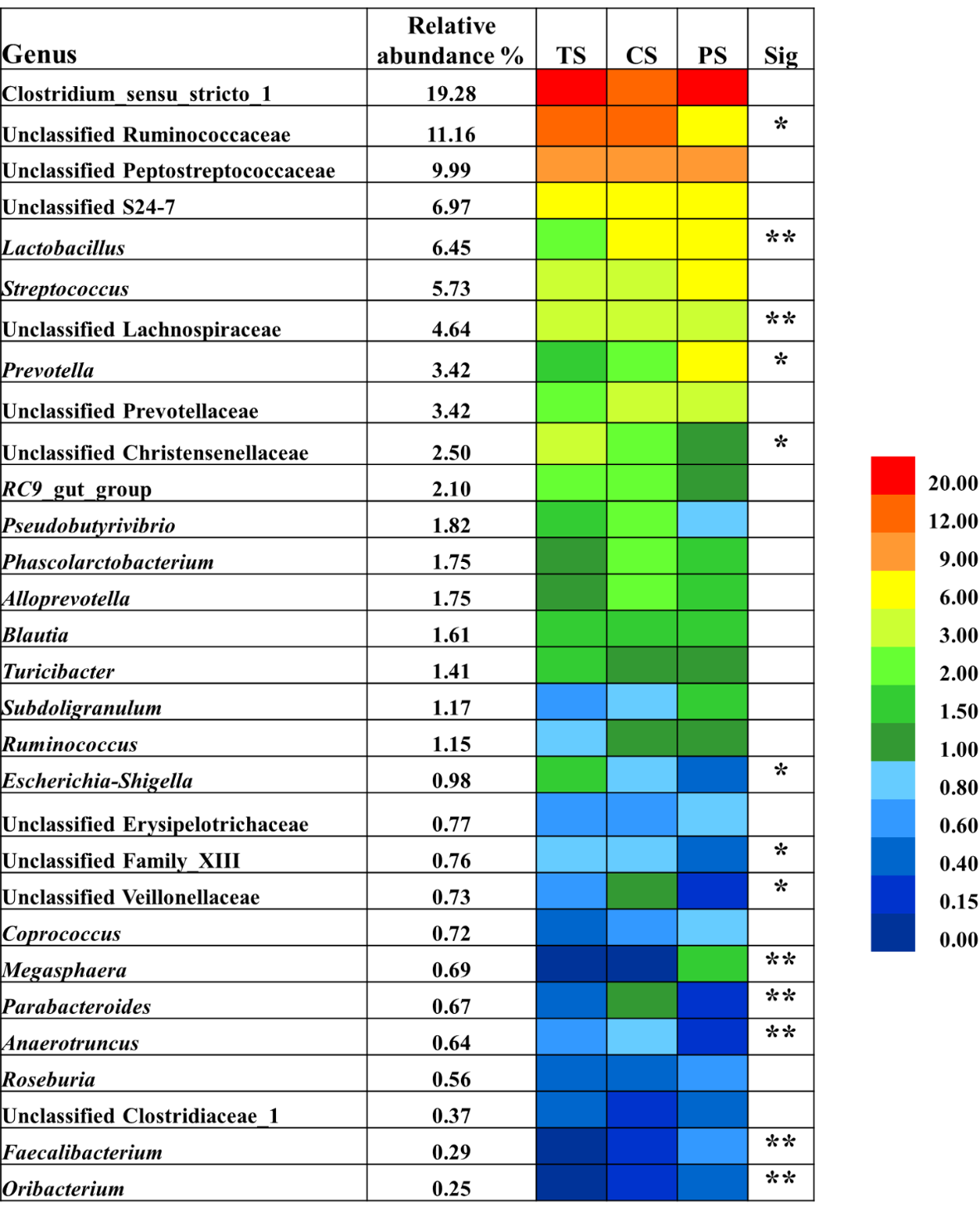

Supplement: Supplementary file 1 [file Data_Sheet_1.docx]
